# Natural Killer Cells and Their Role in Rheumatoid Arthritis: Friend or Foe?

**DOI:** 10.1100/2012/491974

**Published:** 2012-04-01

**Authors:** Hamid Shegarfi, Fatemeh Naddafi, Abbas Mirshafiey

**Affiliations:** ^1^Department of Anatomy, Institute of Basic Medical Sciences, University of Oslo, 0316 Oslo, Norway; ^2^Department of Immunology, School of Public Health, Tehran University of Medical Sciences, P.O. Box 6446 Tehran 14155, Iran

## Abstract

Rheumatoid arthritis (RA) is a long-term disease that leads to inflammation of the joints and surrounding tissues. Natural killer (NK) cells are an important part of the innate immune system and are responsible for the first line of defense against pathogens during the initial immune challenge before the adaptive immune system eventually eliminates the infectious burden. NK cells have the capacity to damage normal cells or through interaction with other cells such as dendritic cells, macrophages, and T cells cause autoimmune diseases, such as RA. NK cells isolated from the joints of patients with RA suggest that they may play a role in this disease. However, the involvement of NK cells in RA pathology is not fully elucidated. Both protective and detrimental roles of NK cells in RA have recently been reported. A better understanding of NK cells' role in RA might help to develop new therapeutic strategies for treatment of the RA or other autoimmune diseases. We have decided in this paper to focus on the NK cell biology, and attempt to bring the interested readership of this Journal up to date on the NK cell, specifically its possible relation to RA.

## 1. Introduction

Rheumatoid arthritis (RA) is a chronic inflammatory disease determined by an inflammation of the synovial membrane leading to destruction of cartilage and bone [1]. The interaction between genetic and environmental factors can contribute to RA occurrence [2]. RA is more prevalent among women than men [3]. It has been demonstrated that osteoclasts are crucial mediators of all forms of bone loss in RA [4]. TNF-*α* induces synovial fibroblasts and macrophages (MØs) to release IL-1. TNF-*α*, IL-1 and, RANKL promote osteoclast activation and osteolysis in RA [4]. Recent studies have indicated that HLA-DRB1 SE alleles are associated with a severe course of RA, and a parameter that can be measured is bone destruction [5]. It has been indicated that RA patients expressing a combination of two SE-associated HLA-DRB1 alleles exhibit the most severe small joint damage in the initial stages of the disease and suffer a high proportion of long-term large joint destruction [6]. Plasma soluble HLA-G levels are lower in RA patients than in controls, and low soluble HLA-G indicates that T and natural killer (NK) cell activities are not inhibited by soluble HLA-G molecules in RA [7]. The accumulation of NK cells has been demonstrated in the synovial fluid in patients with RA [8]. Hence, knowledge of NK cells and NK cell receptors may be of great interest for their role to RA. In this review, we focus on current knowledge regarding NK cells and NK cell receptors in human autoimmune diseases such as RA. 

## 2. Natural Killer Cells

Natural killer (NK) cells were defined by their ability to spontaneously kill tumor cells and virally infected cells [9, 10]. To date, we know that these cells are capable of recognizing and destroying a wide variety of target cells, including transplanted, virally infected, antibody-coated, stressed, and transformed cells [11]. NK cells constitute the third major population of lymphocytes together with T and B cells. The majority of NK cells are believed to be relatively short lived, although more long-lived subpopulations of NK cells have been identified in lymph nodes (LNs) and thymus [12]. There are about 2 billion NK cells in adults and they are mostly found in the blood, bone marrow (BM), spleen, liver, LNs, thymus, lung, peritoneum, and in the uterus during gestation.

**Figure 1:**
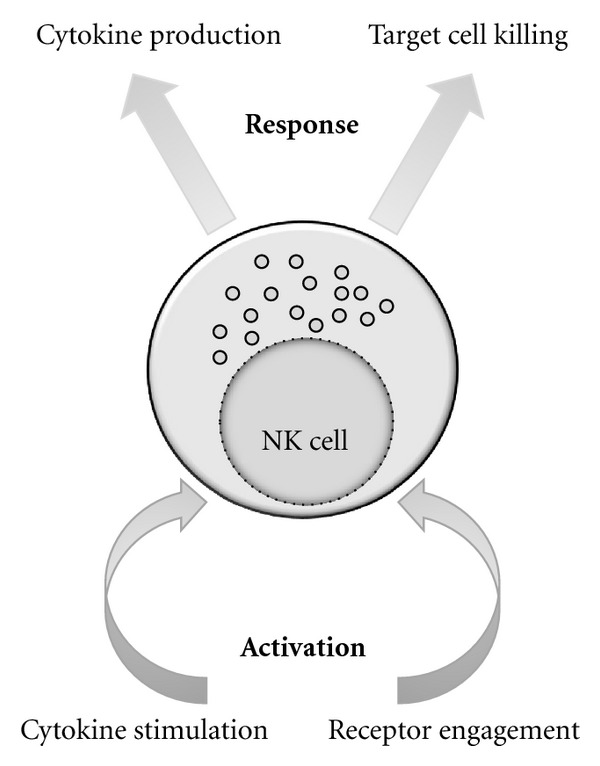
NK cells can be activated by inflammatory cytokines and/or NK-receptor ligand engagement. In turn, they can produce an array of cytokines or directly kill target cell.

 The two major functions of NK cells are cytotoxicity and cytokine production (Figure 1). NK cells display heightened cytotoxicity when activated by cytokines, such as IL-2 or IL-15. NK cells are capable of performing antibody-dependent cellular cytotoxicity (ADCC) through CD16 (low-affinity Fc*γ* receptor IIIA). CD16 binds to the Fc tail of antibodies. When target cells are coated with antibodies, they may induce ADCC. NK cells may kill tumors and virally infected cells through the induction of apoptosis. Perforin is stored in cytoplasmic granules that are released upon NK cell activation. Perforin monomers are inserted into the plasma membrane of target cells and polymerize into a pore through which granzyme A and B enter and induce apoptosis. Perforin is constitutively expressed in NK cells but its expression is enhanced by IL-2 stimulation [12, 13]. NK cells also express TNF-related apoptosis-inducing ligand (TRAIL) and FasL, which are important mediators of apoptosis. TRAIL is only expressed by subpopulations of resting NK cells, but is generally expressed after stimulation by IL-2, IFN-*α*/*β*, or IL-15. Fas is a transmembrane protein expressed by abnormal cells and may induce apoptotic signals after binding to FasL on NK cells [13].

NK cells also produce cytokines, of which IFN-*γ* is critically important both in the innate and adaptive immune responses. It has both immunostimulatory and immunomodulatory effects. It induces T_H_1 responses and upregulates MHC-I expression on a variety of cells, such as antigen presenting cells (APCs). Subpopulations of NK cells may also produce TNF-*α*, GM-CSF, IL-5, IL-13, IL-10, and TGF-*β*. It has been reported that some cytokines, including IL-2, IL-12, IL-15, and IL-18 may stimulate cytokine production by NK cells [11, 13–15].

## 3. NK Cell Definition

NK cells are derived from haematopoietic stem cells in the BM. This is also the place for primary NK cell development. These cells may also develop at peripheral sites such as liver [16]. Peripheral activation of mature NK cells may result in phenotype modification and modulation of NK cell effector functions [14, 16]. In humans, NK cells have been phenotypically defined as CD3^−^CD56^+^ lymphocytes that may be further subdivided into CD56^dim⁡^  CD16^bright^ (90% of all NK) and CD56^bright^ CD16^−^ cells. These subpopulations differ in regard to cytotoxic capacity and cytokine profiles [17]. In mice, the best model for studying NK cells, NK cells are defined as CD3^−^NK1.1^+^CD122^+^ [12].

## 4. NK Gene Complex (NKC) and Leukocyte Receptor Cluster (LRC) 

The human and mouse NKCs are located on chromosomes 12 and 6, respectively. These regions encode most of the NK cell receptors in these species. The murine NKC encodes lectin-like molecules that can be classified into families of highly homologous members. These receptors are encoded by clusters of closely linked genes that usually are separated from the other gene families. Among families, the receptors share some general structural features but display limited homology [18]. In humans, LRC located on chromosome 19 encodes killer cell Ig-like receptors (KIR).

## 5. NK Cell Receptors

Several NK cell receptor families have been identified (Table 1). NK cell functions are controlled by a repertoire of activating and inhibitory receptors. Usually more than one antigen receptor is expressed on a single NK cell. This is in contrast to B and T cells, which are mainly controlled by single antigen receptors (BCR and TCR, resp.). The genes that encode NK receptors do not undergo recombination. The killer immunoglobulin-like receptor (KIR), Ly49 and CD94/NKG2 receptors monitor MHC-I molecules while other families such as the activating NKG2D receptor, the only member of this family, recognize MHC-like molecules; for example, MICA in humans and RAE ligands in mice [13].

 NK cell receptors can be structurally divided in two groups: the killer cell lectin-like receptors (KLR) and immunoglobulin (Ig) superfamily receptors. The former include the NKR-P1 (KLRB), CD94/NKG2 (KLRC/D), and NKG2D (KLRK) receptor families. The latter includes the killer cell Ig-like receptors (KIR), natural cytotoxicity receptors (NCR), and Fc*γ*RIII (CD16). It should be noted that many NK cell receptors are not restricted to NK cells but can also be found on other cells.

## 6. Killer Immunoglobulin-Like Receptor (KIR) Family

Killer cell Ig-like receptors (KIR) which apparently function as MHC-I recognizing receptors akin to Ly49 receptors in rodents encoded by the LRC located on chromosome 19. The KIR family is a highly polymorphic multigene family of receptors. The KIR receptors are classified by the number of extracellular Ig domains (2D or 3D). They have long (KIR-L) or short (KIR-S) cytoplasmic tails. KIR-L contains two ITIMs responsible for their inhibitory function. KIR-S lacks ITIMs, but instead has a charged amino acid in their transmembrane domains necessary for their activating function. KIRs may be expressed as monomers or disulphide-linked homodimers. Like Ly49s, KIRs are expressed by overlapping subsets of human NK cells and some T-cell subsets [11, 19].

 Like Ly49 receptors in rodents, human KIRs recognize allelic variants of HLA class I, HLA-A, HLA-B, and HLA-C, molecules. KIR can discriminate between different peptides presented by HLA molecules; however, these receptors do not distinguish self from nonself peptides [11, 20]. The specific ligands for some inhibitory KIR have been characterized and it has been shown that some of the KIR2D subfamily recognizes a polymorphism in HLA-C proteins while KIR3DL1 reacts with HLA-B and certain HLA-A proteins that possess the Bw4 serological epitope. KIR3DL2 has been shown to recognize certain HLA-A ligands. The specificity of the activating KIRs has been less characterized and, seemingly, they do not bind to HLA class I and if they bind, the affinity is much weaker than that of the paired inhibitory KIR. For instance, the extracellular domain of the activating KIR2DS2 differs from KIR2DL2 and KIR2DL3 by only 3 or 4 amino acids, respectively, and fail to bind to HLA-C*0304 [21].

*KIR2DL4* is the most distinct gene in the KIR family and encodes the activating receptor KIR2DL4 while it contains ITIM in its cytoplasmic domain. In contrast to other activating KIR, it associates with the Fc*ε*RI*γ* adapter protein rather than with DAP12. It has been suggested that the HLA-G is the cognate ligand of KIR2DL4 [11]. As mentioned, HLA-G has been shown to be involved in RA [7].

## 7. NKG2 (KLRC)/CD94 (KLRD) Families

CD94 is expressed together with NKG2A, NKG2C, or NKG2E. NKG2A has two ITIMs in its cytoplasmic tail inferring an inhibitory function to the CD94/NKG2A receptor. The activating CD94/NKG2C/E receptor is associated with the adapter protein DAP12. DAP12 binding is necessary for stable surface expression and receptor signalling. Human CD94/NKG2A and CD94/NKG2C receptors bind to the nonclassical HLA-E molecules while mouse receptors recognize H2-Qa1^b^ [11]. These molecules present peptides from the leader segments of other MHC-I molecules. NK cells in this manner may indirectly monitor the general level of host MHC-I expression [11]. The CD94/NKG2 receptors and their corresponding ligands are relatively conserved. These receptors are expressed by subsets of NK and T cells. Unlike the stably expressed KIR and Ly49 receptors, receptor expression of CD94/NKG2 is modulated by cytokines. As for the KIRs and Ly49s, the inhibitory CD94/NKG2A receptor appears to have higher ligand affinity than its activated counterpart.

## 8. NKG2D Family

The NKG2D receptor is expressed as a homodimer on NK cells, activated CD8^+^ T cells, subsets of *γδ*T cells, NKT cells and in certain conditions, human CD4^+^ T cells [17, 22]. NKG2D has received considerable attention because of its role in immune responses related to cancer, infection, and autoimmunity. NKG2D has little homology with other NKG2 receptors. Expression of NKG2D requires an association with the adaptor proteins DAP10 or DAP12 [11]. In the mouse, two isoforms of NKG2D have been identified. The longer protein (NKG2D-L) exclusively is associated with the DAP10 whereas the short isoform (NKG2D-S) can pair with either DAP12 or DAP10. NKG2D receptors mediate “*induced self-recognition*” that is, it recognizes ligands that are upregulated on stressed or diseased cells [23]. Human NKG2D ligands include the MHC-I-like molecules MICA and MICB and UL16-binding proteins: ULBP1, -2, -3, and -4. In mice the ligands include retinoic acid early transcript 1 (RAET1) ligands: RAE-1*α*, -*β*, -*γ*, -*δ*, and *ε*, H60, and MULT1 [11, 13]. NKG2D was first cloned in humans [24], and later in mice [25].

## 9. Natural Cytotoxicity Receptor (NCR) Family

The stimulatory receptors NKp30, NKp44, NKp46, and NKp80 belong to the natural cytotoxicity receptor (NCR) family [20]. They are involved in tumour surveillance possibly by mediating “*induced self-recognition.*” NKp46 and NKp30 are expressed by all NK cells whereas only activated NK cells express NKp44. NKp44 and NKp80 have not been found in rodents. Ligands for the NCRs may include viral haemagglutinins (NKp46 and NKp44), heparan sulphate proteoglycans (NKp30 and NKp46), the nuclear factor HLA-B-associated transcript 3 (NKp30), and activation-induced C-type lectin (NKp80) [20]. The NCRs are encoded by genes within the LRC which also encodes KIRs in humans. The stress-induced B7-H6 molecules expressed by human tumor cells may trigger NKp30-mediated activation of NK cells [26]. The NKp46 which plays an important role in killing cancer cells [27] and virally infected cells [28] has been characterized in humans, rodents, monkeys, and cattle [29]. NKp46 is exclusively expressed by NK cells, and is probably the best NK cell marker available. The ITAM present in NKp46 associated with CD3*ζ* and Fc*ε*RI*γ* suggests activating the NK cell cytotoxicity in a similar manner to CD16 [30].

## 10. NKR-P1 (KLRB) Family

The NKR-P1 receptors were initially characterized in the rat [31] but later in mice and humans [32]. They recognize the Clr molecules. NKR-P1A, -B, -F, and -G receptors have been characterized in the rat while NKR-P1A, -B/-D, -C, -F, and -G receptors have been studied in the mouse. NKR-P1A is the only receptor present in humans. It is an inhibitory receptor and shares 45% amino acid identity with the mouse NKR-P1 molecules. It is expressed by subsets of NK and T cells [33].

## 11. KLRG Family

KLRG1 or mast cell function-associated Ag (MAFA) belongs to the C-type lectin-like superfamily and is an ITIM-containing receptor. In mice and humans, this well-conserved receptor is found on subsets of T and NK cells. Ligands for KLRG1 are E-, N-, and R-cadherin. Expression of these ligands may be lower in metastatic tumours rendering these cells more sensitive to NK-mediated killing [34]. Expression of KLRG1 increases substantially in T and NK cells during viral, bacterial, and parasitic infections in mice. KLRG1 is also expressed on FoxP3^+^ regulatory T cells [35].

## 12. Other NK Cell Receptors

Fc*γ*RIII (CD16) is expressed by NK cells, MØ, neutrophils, and mast cells. It binds to the Fc (fragment, crystallisable) portion of the human IgG1 and IgG3 antibodies. Antibody binding induces ADCC, an effector mechanism that NK cells employ to kill antibody-coated target cells. However, some viruses, such as flaviviruses exploit Fc receptors to infect cells, a mechanism known as antibody-dependent enhancement of infection [11]. Signalling *via* CD16 may cause not only degranulation and cytokine production, but also apoptosis of NK cells [36]. 2B4 is a member of the signalling lymphocyte activation molecule-(SLAM-) related family of receptors and is expressed by all NK cells, *γσ*T cells, subsets of CD8^+^ T cells and human monocytes [33]. CD48 is the ligand for 2B4. Mouse 2B4 may function as an activating or inhibitory receptor depending on its splice forms while human 2B4 is an activating receptor [33]. The DNAX accessory molecule 1/CD226 (DNAM-1) receptor is expressed by most NK cells, some B and T cells, DC, monocytes, and thrombocytes. It binds to CD155 and CD112 (nectin-2) present on cancer cells surface and endothelial cells and may be important for extravasation of NK cells [11].

## 13. NK Cell Signal Transduction

NK cell functions are controlled by inhibitory and activating receptors. The inhibitory receptors often have ITIMs in their cytoplasmic tails while most activating receptors noncovalently are associated with ITAM containing adaptors. Binding to these adaptors may elicit downstream signalling events leading to cytoskeleton rearrangements, proliferation, secretion of lytic granules and cytokines by activating-bearing NK cells and T cells. ITAMs have the consensus sequence (D/E)xxYxx(L/I)x_6-8_Yxx(L/I) where x denotes any amino acid and slashes separate alternative amino acids. The adapters have a negatively charged amino acid in their transmembrane segment that associates with a corresponding positively charged amino acid (arginine or lysine) in the receptor. Receptor ligation leads to phosphorylation of the ITAMs. This recruits and activates Syk or ZAP-70 tyrosine kinases that activate signalling cascades and ultimately lead to cellular activation. NK cells express three ITAM-bearing adaptor proteins: CD3*ζ* (having three ITAMs per chain), Fc*ε*RI*γ* and DAP12 (both having single ITAMs). Fc*ε*RI*γ*, and CD3*ζ* are expressed as either disulphide-linked homodimers or heterodimers whereas DAP12 is exclusively found as a disulfide-linked homodimer [36]. DAP10 shares little homology (20%) with DAP12 and is expressed as a disulfide-linked homodimer and has a cytoplasmic tail with an YxxM motif. This is a potential Src homology 2 (SH2) domain-binding site for the p85 regulatory subunit of the phosphatidyl-inositol (PI) 3-kinase [11, 13].

 Inhibitory receptors often override signals that are generated by activating receptors, and binding these receptors to their ligands on target cells results in suppression of cytotoxicity and cytokine secretion by NK cells. Inhibitory receptors usually contain one or more ITIMs (I/L/V/S)x/Yxx(L/V) (x represents any amino acid) [36]. After engagement of the inhibitory receptor, the tyrosine residue of the ITIM is phosphorylated probably by Src family kinases. The ITIM may then recruit SH2-containing protein tyrosine phosphatises, such as SHP-1, SHP-2, or SHIP [36]. These phosphatases may then shut down cell activation. Many inhibitory NK receptors recognize MHC-I molecules. Cells expressing normal levels of MHC-I may in this manner be protected against NK cell killing [36, 37].

## 14. NK Cell Migration or Trafficking

NK cells secrete several chemokines, including CCL3, CCL4, and CCL5 (RANTES) [38] and express an array of chemokine receptors [39, 40]. Consistent with their role in immune surveillance, NK cells are widely distributed. Mouse NK cells are found most frequently in the lung and liver (lung > liver > blood > spleen > BM > LNs > thymus) [40]. NK cells also appear to be frequent in nonlymphoid organs, such as the epithelium of the gut [15, 41] and in the uterus during pregnancy [42]. NK cell functions and receptor profiles differ widely depending on their tissue localisation. Whether these alterations in NK cells properties are a consequence of having different homing capacities or an underlying cause of their tissue-dependent maturation is unclear. NK cells may be recruited to various tissues upon inflammation. The mechanisms governing NK cell trafficking remain poorly understood [40].

## 15. Regulation of NK Cell Effector Functions and Tolerance

NK cells have the capacity to damage normal cells. It is important to keep NK cells in check in the normal situation. NK cells may lyse cells lacking one or more self-MHC-I molecules. Engagement of self-MHC-I molecules by inhibitory NK receptors may be the principal mechanism by which killing of normal cells is prevented. There are still many unresolved questions regarding NK cell tolerance. Such knowledge needed to understand the role of NK cells in autoimmunity, tumour surveillance, stem-cell transplantations, and antiviral responses [23, 43–45].

 The “*missing self*” hypothesis proposed by Kärre and colleagues in 1986 suggested that NK cells monitor cells for normal MHC-I expression by inhibitory NK receptors [46]. Virally infected cells and tumor cells often downregulate MHC-I expression in order to evade CD8^+^ T-cell recognition, but this may render them sensitive to NK mediated killing. In the absence of inhibitory ligands, NK-cells may become activated through stimulatory receptors and thus kill MHC-I-deficient cells. Alternative “*missing self*” mechanisms may also exist. Inhibitory NKR-P1 receptors may prevent killing of cells expressing Clr ligands. The 2B4 receptor may prevent killing of CD48 expressing target cells [33]. Conversely, high-level expression of activating ligands may lead to NK cell activation even in the presence of inhibitory ligands. This has been shown for the NKG2D receptor [47].

 NK cells from MHC-I-deficient mice have been shown to be “*hyporesponsive*” [43, 48]. However, these cells can become fully functional when transferred to an MHC sufficient environment [49, 50]. Furthermore, the expression of NK receptor surface is often downmodulated in the presence of ligands. This has been clearly demonstrated for Ly49 receptors; a phenomenon referred to as “*receptor calibration*” [51, 52]. Different models explaining NK cell self-tolerance and “*education*” has been proposed [47, 53]. In the “*at least one receptor model*” proposed by Peter Parham, every NK cell with killing capacity must express at least one inhibitory receptor for self MHC-I molecules. Once engaged these mediate inhibitory signals that prevent NK-mediated killing of autologous cells. These interactions may also play a critical role in NK-cell maturation [16, 48]. According to the “*arming*” [44] or “*licensing*” hypothesis these inhibitory receptors allow NK cells to be fully functional [43, 45]. NK cells that are unable to come up with the appropriate inhibitory receptors are left in an “off” or “*uneducated*” state incapable of effectively recognizing and killing target cells. Furthermore, it has been argued that NK-cell “*education*” is a quantitative and dynamic process depending on which MHC-I and inhibitory receptors involved [47]. However, NK cell tolerance may be more complex since a subset of mouse NK cells lacking inhibitory MHC-I receptors have been shown to be functional [54, 55]. Therefore, alternative mechanisms for NK tolerance may exist [33, 48, 53].

## 16. NK Cells and Memory Cells

Immunological memory has been thought to be present only in the adaptive immune system. Recently “adaptive” memory-like NK cells have been described; first in a model for hapten-induced contact hypersensitivity (CHS) [56]. Later it was shown that Ly49H^+^ NK cells selectively proliferate and persist in mice for several months after CMV infection [57]. Upon reinfection, these mice created faster and stronger NK cell responses than naïve mice. Interestingly, transfer of low numbers of these adaptive NK cells into naïve neonatal mice resulted in greater protective immunity than that of naïve mature NK cells [57, 58].

## 17. NK Cells and Diseases

NK cells through their functions, cytotoxicity, and cytokine production, can act as an immune regulator. To date, the extremely rare cases of selective NK cell deficiencies in humans have been reported. This makes it difficult to elucidate *in vivo* the role of NK cells in the onset and/or progression of autoimmune diseases. However, several NK cell deficits have been described. Most of these can be attributed to broader immunological defects like caspase 8 deficiency, TAP-2 deficiency, and the DAP12-deficient form of Nasu-Hakola disease. A few examples of isolated NK cell deficiencies have been described. NK cell deficiencies in humans result in overwhelming fatal infection during childhood, in particular herpes virus infections [59, 60]. NK cells may also participate in immunity to HIV [61] and cancer [17].

## 18. NK Cells and Autoimmune Disease, RA

It has been suggested that NK cells have a disease-promoting or a disease-controlling role in autoimmune diseases. Here, we review some reports related to rheumatoid arthritis and our question is that: are NK cells involved in Rheumatoid arthritis disease?

 The role of NK cells in RA is not clear. Several reports have indicated that NK cells may have direct or indirect role in RA [62]. Some of these reports have characterized NK cells in RA tissue with disease-promoting functions. Dalbeth and Callan reported that a subset of NK cells (CD56^bright^) is greatly expanded within inflamed (synovium) joints [63], in which they produce more IFN-*γ* compared with the blood NK cells from the same patients [64]. Moreover, these NK cells could induce the differentiation of monocytes into DCs. The communication between NK cells and other cell types through cytokines and chemokine actually is a potential risk for autoimmune diseases. Other example of this phenomena is the crosstalk between NK cells and myeloid DCs, referred to as “DC editing,” which may lead to NK cell activation and DC maturation. In this way, activated NK cells may in turn kill immature DCs that fail to undergo proper maturation [65]. Furthermore, it has been reported that NK cells can function as APCs in some instances, which complicate the involvement of these cells in the immune responses [66].

## 19. Genetic Background

Studies of possible genetic risk factors that link NK cell receptor genes to RA are preliminary. However, there is clear evidence that KIR is implicated in human autoimmune disorders. Yen et al. have found that patients with RA complications have an expansion of unique population of CD4^+^CD28^−^ T cells which is uncommon in healthy individuals [67]. This cell population is potentially involved in endothelial damage. Interestingly, CD4^+^CD28^−^ T cells are functionally distinct from classical CD4^+^  T_H_ cells and share some features with NK cells. For instance, they do not express CD40 ligand, but express CD57 (an NK cell marker), and produce large amounts of IFN-*γ*, and produce granzyme B and perforin [67]. The authors further focus on T cell subsets which express the activating KIR molecule, KIR2DS2, in the absence of DAP12. Therefore, signalling in these T cells could be deviated and mediates autoimmune disease [68]. Furthermore, they have shown that individuals possessing a *KIR2DS2* gene and certain *HLA-C* alleles are more exposed to RA with vascular complications than healthy individuals or arthritis patients without vascular complications.

 These studies also indicate that expression of activating KIR, in the absence of an inhibitory receptor for self MHC-I, may contribute to autoimmune disorders. In this case, the activating KIR expressed in effector T cells may synergize with the signals transduced by TcR, otherwise insufficient for an autoantigen alone, to elicit an autoimmune response [11].

## 20. The Role of NK Cells in Immunopathogenesis of RA

It has been suggested that NK cells can play both a protective and a pathogenic role in rheumatoid arthritis [69]. The interplay between NK cells and other cells of natural and specific immunity will occur through release of cytokines. One of the most potent osteoclastogenic cytokines which is pivotal in the pathogenesis of RA is TNF-*α* [12]. TNF-*α* induces receptor acquisition by NK cells and the combination of TNF-*α* and IL-15 can enhance this effect [70]. TNF-*α* has a role in NK-dependent DC maturation [71]. Although MØs and monocytes are the major producers of TNF-*α* in RA, T cells are abundant in RA synovium, and both CD4^+^ and CD8^+^ T cells can produce large amounts of TNF-*α* and TNF-*β* [72]. As mentioned above, CD28^−^CD4^+^ T cells can express the NK cell receptors KIRs and CD57 [73].

 It has been demonstrated that NF-*κ*B is an important factor in regulation of NK cell growth and differentiation. NF-*κ*B is activated in the presence of TNF-*α* plus IL-15 [70]. NF-*κ*B signalling pathways can mediate crucial events in the inflammatory response by chondrocytes, leading to extracellular matrix damage and cartilage destruction [74]. NK cells can cause DC maturation during the innate phase of the immune response and membrane-bound IL-15 on DC surface seems to be essential for NK cell proliferation and survival [71]. NK cells produce IFN-*γ* which is induced by DC-derived IL-12 and IFN-*γ* can act as a synergistic or regulatory factor for DC maturation. Soluble factors as well as cell-to-cell contact have a roll in NK cell activation by DCs. Type 1 IFNs, IL-12, and IL-15 have been shown to be crucial in mature DC-dependent NK cell activation [71]. It is demonstrated that coculture of NK cell and DCs leads to DC maturation, production of TNF-*α* and IL-12 and also upregulation of ligands, such as CD86. In addition, IFN-*γ* can upregulate the NKG2D ligands MIC A/B on monocyte-derived DCs and these molecules activate NK cells in a cell contact-dependent fashion [71]. IL-15 is thought as one of the major instigators of RA pathogenesis, together with other cytokines, such as TNF-*α*, IL-6, and IL-18 [75]. IL-6 has an important role in rheumatoid inflammation [76]. It can act synergistically with IL-15 to enhance the cytotoxic activity of NK cells [77]. IL-15 is produced by infected MØ and it can induce IFN-*γ* production, NK activation, and enhanced TNF-*α* production by T cells [78]. It can activate CD57^+^CD4^+^ cells to induce TNF-*α* production from monocytes [73]. IL-15 may have a role in initial stage of osteoclastogenesis. It has been demonstrated that osteoclastogenesis may occur through the expression of PLD1-induced RANKL in rheumatoid synovial fibroblasts stimulated by IL-15 [79]. Both IL-15 and IL-2 can have same functions such as activation of T cells and stimulation of NK cell proliferation. Levels of IL-15 will increase with RA disease duration in the serum and synovial membrane [80]. IL-18 released by DCs and MØs induces NK cells to synthesize IFN-*γ* which acts with IL-12 and IL-15 to shift T cells toward the T_H_1 profile. Moreover, IL-18 can cooperate with IL-2 to induce the T_H_2 profile [78]. Thus, selective manipulation of T_H_ cell differentiation to induce T_H_2 effectors can be an effective approach for interrupting ongoing and established T_H_1-driven chronic inflammatory diseases, such as RA [81].

 The combination of IL-18 and IL-12 can increase NK activity in both knockout and wild-type controls. NK cells can be an immediate source of both latent and active TGF-*β* [82]. IL-2 can up-regulate the production of active TGF-*β*, and TNF-*α* has a positive effect on TGF-*β*. The combination of IL-2 and TNF-*α* has additive effects on TGF-*β* [82]. It has been shown that IL-2 prevents the TGF-*β*-induced NKG2D downregulation in NK cells via the JNK pathway [83]. IL-10 can decrease the production of active TGF-*β*. Recent studies indicate that IL-17 is overexpressed in RA patients and IL-10 suppresses IL-17 expression. Thus, IL-10 may be useful in the treatment of autoimmune diseases [84].

 IL-22 is an immune mediator, which is produced by activated T and NK cells. IL-22 can amplify the effects of TNF-*α*, IFN-*γ*, and IL-17 [85]. It has been proved that human T_H_1 cells are the most important IL-22 producers. T_H_17 and T_H_22 are demonstrated to be important IL-22 producers, but in humans T_H_22 and T_H_1 cells play a more prominent role for IL-22 production. TGF-*β* can downregulate the IL-22 production capacity of T_H_17 in both the human and mouse. TGF-*β* inhibits the development of T_H_22 cells [85]. IL-22 production by T_H_17 cells has been indicated to be dependent upon IL-23. Expression of IL-22 can be upregulated in synovium in RA and IL-22 can induce synovial fibroblast proliferation and chemokine production [86]. The level of IL-22 has been increased in the serum of half of the patients with RA. Thus, it indicates a possible involvement of IL-22 in the pathophysiology of RA [87].

 For many years, it has been discussed that T_H_17 is responsible for collagen-induced arthritis (CIA) as experimental model of RA in animals. Moreover, in CIA mice it has been shown that NK cells suppress T_H_17 cell development by the production of IFN-*γ* [88]. The involvement of T_H_1 cells in pathogenesis of RA cannot be ruled out, especially since studies in an animal model of arthritis different from CIA have indicated that IFN-*γ* is essential for disease development [88]. On the other hand, IL-1 can also activate monocytes, MØs, and NK cells, and it is produced by various cell types, such as MØ, monocytes, and synovial lining cells. These cell types can produce inflammatory mediators, such as IL-1, TNF-*α*, IL-6, and IL-8. These mediators are responsible for infiltration of inflammatory cells into inflammatory sites, increase in blood vessels permeability and induction of fever. IL-1 can activate synovial cells and osteoclasts to produce metalloproteases and collagenases that cause destruction of cartilage and bone [89].

 Both the NK cell activity and the activity on a per-cell basis are reported to decrease in RA cases. The expression of NKG2D, CD16, and CD244  receptors also decreases in RA patients indicating that a low NK activity on a per-cell basis can contribute to an impaired NK activity in RA patients [64]. All these observations suggest that NK cells directly or indirectly are involved in the complex processes of RA.

## 21. Conclusions

Rheumatoid arthritis is a chronic autoimmune disease characterized by joint inflammation and bone destruction. Excessive cytokine production driven by cell-cell interactions within the joint contributes to the disease progression. NK cells are prominent components of the innate immune response and because of their ability to secrete a variety of cytokines they could have a disease-promoting or a disease-controlling role in autoimmune diseases including RA. Our understanding of NK cell is recently much improved; however, many aspects of NK cell biology still are unexplained and unexplored. Nevertheless, further careful analysis and studies of how NK cells communicate with dendritic cells, macrophages, and T cells will contribute to a better understanding of their role in autoimmune diseases including RA. This knowledge might allow the development of new therapeutic strategies based on NK cells for the treatment of RA.

## Figures and Tables

**Table 1:** NK cell receptors can be divided in two groups based on binding to MHC class I- or non MHC class I molecules as their ligands. NK cell receptors can also be structurally divided in two groups as immunoglobulin superfamily or killer cell lectin-like receptors.

NK cell receptors			
MHC-I binding receptors		Non MHC-I binding receptors	
	Species		Species
Ly49 family	Mouse	Ly49 family	Mouse
KIR family	Human	NKR-P1 family	Human and mouse
CD94/NKG2 family	Human and mouse	NCR family	Human and mouse
		NKG2D family	Human and mouse

Immunoglobulin superfamily receptors		Killer cell lectin-like receptors	

KIR family		Ly49 family	
NCR family		NKR-P1 family	
Fc*γ*RIII, 2B4, DNAM-1		NKG2D family, CD94/NKG2 family	
